# Association of biomarkers related to preoperative inflammatory and coagulation with postoperative in-hospital deaths in patients with type A acute aortic dissection

**DOI:** 10.1038/s41598-021-98298-w

**Published:** 2021-09-21

**Authors:** Ming Li, Suochun Xu, Yang Yan, Haichen Wang, Jianjie Zheng, Yongxin Li, Yongjian Zhang, Junjun Hao, Chao Deng, Xinglong Zheng, Miaomiao Liu, Yang Gao, Xue Wang, Li Xue

**Affiliations:** 1 Department of Cardiovascular Surgery, The First Affiliated Hospital of Xi’an Jiaotong University, Xi’an, 710061 Shaanxi China; 2 Department of Clinical Laboratory, The Second Affiliated Hospital of Xi’an Jiaotong University, Xi’an, 710004 Shaanxi China

**Keywords:** Cardiology, Diseases, Health care

## Abstract

The aim of this study was to analyze the role of blood biomarkers regarding preoperative inflammation and coagulation in predicting the postoperative in-hospital mortality of patients with type A acute aortic dissection (AAD). A total of 206 patients with type A AAD who had received surgical treatment were enrolled in this study. Patients were divided into two groups: the death group (28 patients who died during hospitalization) and the survival group (178 patients). Peripheral blood samples were collected before anesthesia induction. Preoperative levels of D-dimer, fibrinogen (FIB), platelet (PLT), white blood cells (WBC) and neutrophil (NEU) were compared between the two groups. Univariable and multivariable logistic regression analysis were utilized to identify the independent risk factors for postoperative in-hospital deaths of patients with type A AAD. Receiver operating characteristic (ROC) curve were used to analyze the predictive value of these indices in the postoperative in-hospital mortality of the patients. Univariable logistic regression analysis showed that the *P* values of the five parameters including D-dimer, FIB, PLT, WBC and NEU were all less than 0.1, which may be risk factors for postoperative in-hospital deaths of patients with type A AAD. Further multivariable logistic regression analysis indicated that higher preoperative D-dimer and WBC levels were independent risk factors for postoperative in-hospital mortality of patients with type A AAD. ROC curve analysis indicated that application of combining FIB and PLT could improve accuracy in prediction of postoperative in-hospital mortality in patients with type A AAD. Both preoperative D-dimer and WBC in patients with type A AAD may be used as independent risk factors for the postoperative in-hospital mortality of such patients. The combination of FIB and PLT may improve the accuracy of clinical prognostic assessment.

## Introduction

Type A acute aortic dissection (AAD) is more likely to cause death if it was not diagnosed early and treated appropriately^1^. Currently, both inflammation and blood coagulation have been recognized to be involved in the pathogenesis of type A AAD. As prognostic indicators of patients with type A AAD, various biomarkers relating to inflammation and coagulation have attracted more and more attention, since the application of these indicators predicting the in-hospital mortality of patients with type A AAD may help guide clinical treatment^2–4^.

In the acute phase of AAD, white blood cells (WBC), C-reactive protein (CRP), fibrinogen (FIB), platelet (PLT) and D-dimer are produced or consumed in large quantities. Previous studies were mainly focused on evaluating the prognosis of type A AAD patients using an individual biomarker, which was always difficult to achieve an ideal predictive effect. At present, a new study showed that AAD is a disease of inflammation and coagulation disorders caused by synergy of multiple factors^5^.

In this study, we retrospectively analyzed clinical data of 206 patients with type A AAD to investigate the association between biomarkers relating to preoperative inflammatory and coagulation and postoperative in-hospital mortality of patients with type A AAD. This study will provide objective evidence for early identifying risk factors and assessing the prognosis in surgical treatment of type A AAD.

## Method

### Patients

Patients with Stanford type A AAD who had been admitted to the First Affiliated Hospital of Xi’an Jiaotong University from January 2018 to October 2020 were screened. These patients were diagnosed with type A AAD by the computer tomography. The primary inclusion criteria were patients with Stanford type A AAD within 2 weeks after symptom onset, aged 18 to 75 years. Although there had been total 231 patients with type A AAD being admitted to our department during the period from January 2018 to October 2020, only 206 patients who had received surgical treatment were enrolled in this study. The other 25 patients didn’t undergo timely surgery and eventually died due to medical history, serious complications and other reasons. These 25 patients didn’t conform to the requirement and were not enrolled in this study. Therefore, the exclusion criteria were as follows: (1) History of cardiogenic shock or pericardial tamponade; (2) Traumatic aortic dissection; (3) Iatrogenic aortic dissection; (4) Severe valvular diseases; (5) Congenital heart diseases; (6) Severe organ dysfunctions such as liver and kidney failure; (7) Malignant tumors; (8) Suspected subclinical myocardial involvement (e.g., history of chronic inflammation or acute infections). This study was approved by the Research Ethics Committee of the First Affiliated Hospital of Xi'an Jiaotong University and the written informed consent was given by all subjects. All data was analyzed in the blinded manner. All the study protocols were followed in the guidelines of the Research Committee of Human Investigation of Xi'an Jiaotong University Health Science Center.

### Surgical method

All the patients with type A AAD were treated with surgeries within 1 day after admission. After successful general anesthesia, a patient was placed in supine position, and the right axillary artery was dislocated for use. The patient had a midthoracic incision, and the chest was opened layer by layer to dislocate the innominate vein and brachiocephalic vessels. The patient was treated with pericardial incision, and intubation was implanted in right axillary artery with right atrial to establish extracorporeal circulation. A stent-graft (MicroPort Medical Company Limited, Shanghai, China) and 4-branched prosthetic graft (Vascutek Limited 4 Branch Graft, Newmains Avenue, Inchinnan, Renfrewshire, Terumo) were used in total arch replacement combining with stented elephant trunk implantation. Patients underwent a median sternotomy and total cardiopulmonary bypass. And cannulation of the right axillary artery was used for cardiopulmonary bypass and selective cerebral perfusion. The arterial line was bifurcated for the right axillary artery and for antegrade perfusion via 1 limb of a 4-branched prosthetic graft. During the cooling phase, the ascending aorta was clamped. The proximal ascending aorta was longitudinally opened, and antegrade perfusion of cold-blood cardioplegic solution was directly infused into the coronary ostia. Aortic root procedures were done, and ascending aortic replacement or Bentall was performed depending on the condition of the aortic valve. Circulatory arrest was established when the nasopharyngeal temperature reached 24℃. Selective cerebral perfusion was started through the right axillary artery and Left common carotid artery followed by the opening of ascending aorta and transverse arch. The primary intimal tear in the proximal descending aorta was sealed using the stented elephant trunk, and the distal aorta was transected circumferentially close to the proximal margin of the origin of the left subclavian artery to avoid recurrent laryngeal nerve injury. The stented elephant trunk was then inserted into the true lumen of the descending thoracic aorta in a bound, compressed state. The proximal edge of the residual aorta was trimmed to match the proximal end of the stent graft. The anastomosis between the 4-branched prosthetic graft and the distal aorta containing the intraluminal stented graft was carried out using the ‘‘open’’ aortic technique. After the anastomosis was completed, blood perfusion of the lower body was initiated via the limb of the 4-branched prosthetic graft. One limb of the prosthetic graft was then anastomosed to the left common carotid artery in an end-to-end fashion. After the anastomosis was accomplished, selective cerebral perfusion was discontinued. Then cardiopulmonary bypass was gradually resumed to normal flow and rewarming was started. The innominate and left subclavian arteries were anastomosed to the respective limbs of the 4-branched prosthetic graft in an end-to-end style. The proximal segment of the left subclavian artery was oversewed with a continuous suture. Right heart bypass was performed with residual aortic wall and pericardium after opening circulation.

### Data collection

Clinical data of the enrolled patients was obtained by consulting their medical records. Demographic information, medical history, vital signs, laboratory test and clinical results of the patients were recorded.

### Endpoints

The study endpoint was defined as all-cause deaths during hospitalization.

### Statistical analysis

IBM SPSS Statistical Software 19.0 (SPSS Inc., Chicago, IL, USA) was used for statistical analysis. Continuous variables with the normal distribution were presented as mean values ± standard deviation. Non-normally distributed data were presented as median (interquartile range). Qualitative variables were expressed as frequencies or percentage. The differences between the two groups were analyzed by independent sample t-test, and chi-square test was used for the qualitative variables. The prognostic factors of patients with type A AAD were identified using univariable and multivariable logistic regression analysis. Receiver operating characteristic (ROC) curve was used to analyze the predictive value of D-dimer, FIB, PLT, WBC, neutrophil (NEU) and CRP on prognosis of the patients. *P* < 0.05 was considered to be statistically significant.

### Consent for publication

Our study does not contain any individual person’s data in any form. All authors signed a consent form for publication in case of acceptance.

## Results

### Baseline characteristics of the patients

206 patients were included in this study. Each patient received a surgical operation. Clinical features of all the patients were summarized in Table 1. In our study, there were no significant differences regarding gender, hypertension, dyslipidemia, diabetes, smoking, alcohol abuse, hemoglobin, ALT, AST, EF, and Cr between the death group and the survival group. Furthermore, the indicators including D-dimer, FIB, WBC, NEU, PLT and CRP in the death group were significantly different from those in the survival group. Additionally, no significant difference in surgical time, CPB time, cross-clamp time and HCA time was observed between the two groups.

**Table 1:** Baseline characteristics between the death group and the survival group.

Variable	Non-survivor (n = 28)	Survivor (n = 178)	*P* value
Age (years)	52.3 ± 12.38	51.84 ± 10.88	0.842
Female/Male	5/23	32/146	0.770
Hypertension, n (%)	11 (39.3)	87 (48.9)	0.123
History of smoking, n (%)	10 (35.7)	65 (36.5)	0.590
Alcohol consumption, n (%)	6 (21.4)	35 (19.7)	0.671
Diabetes mellitus, n (%)	1 (3.6)	5 (2.8)	0.911
ALT (U/L)	32.31 ± 27.11	30.76 ± 20.23	0.130
AST (U/L)	39.58 ± 25.42	38.97 ± 21.35	0.074
BUN (mmol/L)	7.4 ± 2.96	6.78 ± 2.52	0.242
Cr (umol/L)	69–139	55–95	0.081
Mb (ng/mL)	67–950	45–152.25	0.071
EF (%)	51.25 ± 3.47	51.82 ± 2.75	0.788
D-Dimer (mg/L)	23.04 ± 17.69	12.66 ± 11.38	0.040
INR	1.23 ± 0.54	1.15 ± 0.21	0.444
FDP (mg/L)	69.08 ± 26.74	42.84 ± 26.59	0.069
FIB (g/L)	2.12 ± 1.51	2.93 ± 1.72	0.041
Cys-C (mg/L)	1.14 ± 0.66	0.91 ± 0.35	0.085
Hb (g/L)	130.12 ± 30.24	133.91 ± 14.65	0.508
WBC (× 10^9^/L)	13.96 ± 4.76	11.53 ± 4.32	0.046
NEU (× 10^9^/L)	11.93 ± 3.70	9.98 ± 4.17	0.024
PLT (× 10^9^/L)	136.25 ± 64.31	174.31 ± 61.75	0.003
CRP (mg/L)	34.64 ± 23.96	29.18 ± 24.31	0.041

Data are mean ± SD, or median (interquartile range), n (%).

ALT, alamine aminotransferase; AST, aspartate transaminase; BUN, blood urea nitrogen; Cr, creatinine; Mb, myoglobin; EF, ejection fraction; INR, international normalized ratio; FDP, fibrinogen degradation product; FIB, fibrinogen; Hb, hemoglobin; Cys-C, Cystatin C; WBC, white blood cells; NEU, neutrophil; PLT: platelet; CRP, C reactive protein.

### ROC analysis

The predictive value of D-dimer, FIB, PLT, CRP, WBC and NEU for in-hospital deaths was evaluated by using the receiver operating characteristic (ROC) method, as shown in Table 2. The area under the ROC curve (AUC) of D-dimer was 0.647 [95% (0.573, 0.716)], and the sensitivity and specificity was 63.0% and 65.2%, respectively (*P* = 0.0128). The AUC of FIB was 0.634 [95% (0.562, 0.705)], and the sensitivity and specificity was 59.3% and 69.6%, respectively (*P* = 0.0221).The AUC of PLT was 0.684 [95% (0.611, 0.750)], and the sensitivity and specificity was 48.2% and 84.2%, respectively (*P* = 0.0028).The AUC of CRP was 0.542 [95% (0.468, 0.616)], and the sensitivity and specificity was 70.4% and 41.1%, respectively (*P* = 0.4560).The AUC of WBC was 0.641[95% (0.567, 0.710)], and the sensitivity and specificity was 55.6% and 72.8%, respectively (*P* = 0.0109). The AUC of NEU was 0.653 [95% (0.580, 0.721)], and the sensitivity and specificity was 55.6% and 75.3%, respectively (*P* = 0.0128).

**Table 2:** Diagnostic value of D-dimer, FIB, PLT, CRP, WBC and NEU for in-hospital mortality.

Variable	AUC	Cut-off value	SE	95% CI	Sensitivity	Specificity	*P* value
D-dimer (mg/L)	0.647	> 10.3	0.0590	0.573–0.716	0.630	0.652	0.0128
FIB (g/L)	0.636	≤ 2.12	0.0593	0.562–0.705	0.593	0.696	0.0221
PLT (× 10^9^/L)	0.684	≤ 122	0.0615	0.611–0.750	0.482	0.842	0.0028
CRP (mg/L)	0.542	> 11	0.0569	0.468–0.616	0.704	0.411	0.4560
WBC (× 10^9^/L)	0.641	> 13.17	0.0554	0.567–0.710	0.556	0.728	0.0109
NEU (× 10^9^/L)	0.653	> 11.94	0.0532	0.580–0.721	0.556	0.753	0.0041

FIB, fibrinogen; PLT: platelet; CRP, C reactive protein; WBC, white blood cells; NEU, neutrophil.

**Figure 1:**
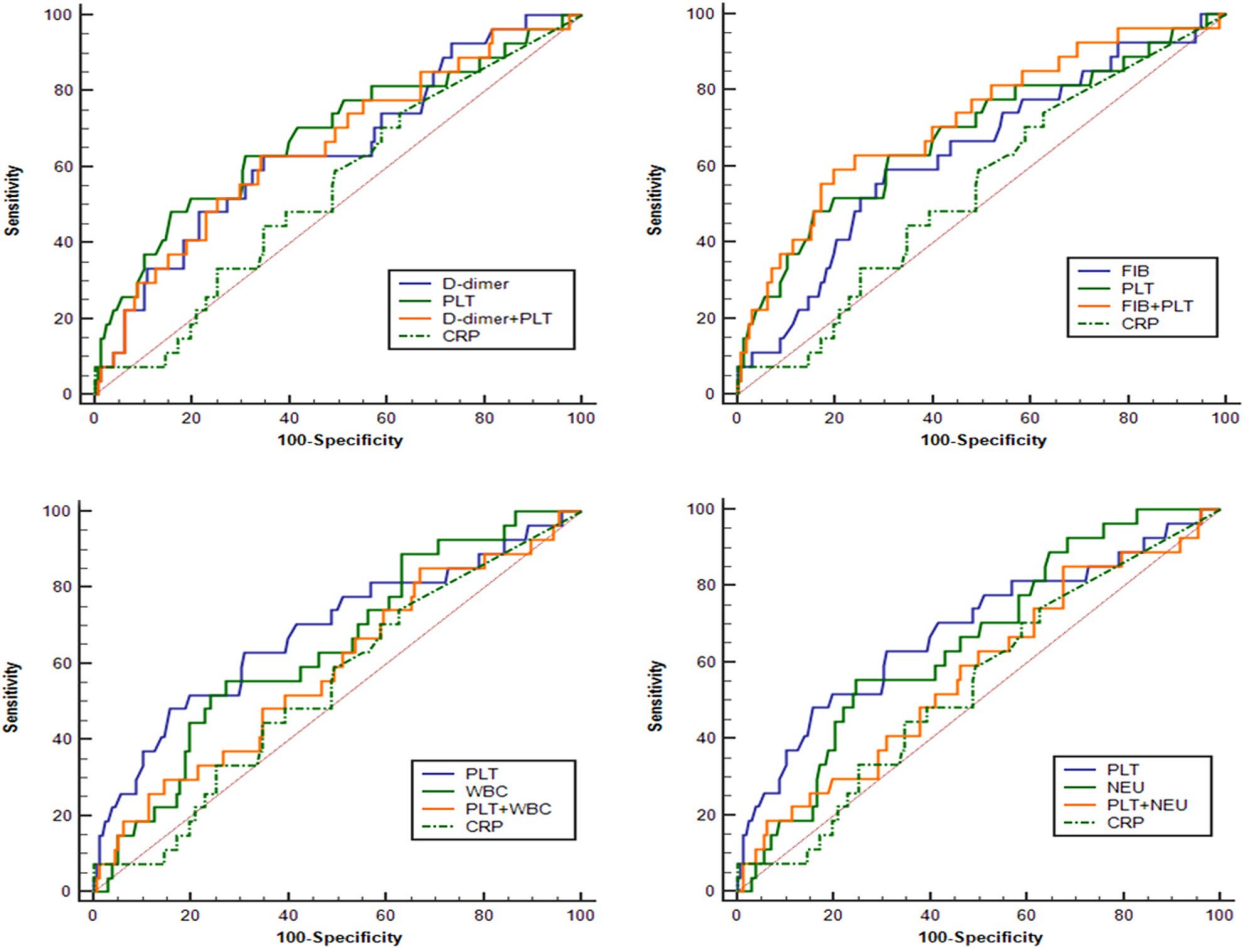
ROC curve of a single index of D-dimer, FIB, PLT, CRP, WBC, or NEU, and the combination of the single index and PLT for predicting in-hospital deaths in patients with type A AAD. ROC, receiver operating characteristic; FIB, fibrinogen; PLT, platelet; CRP, C-reactive protein; WBC, white blood cells; NEU, neutrophil; AAD, acute aortic dissection.

**Figure 2:**
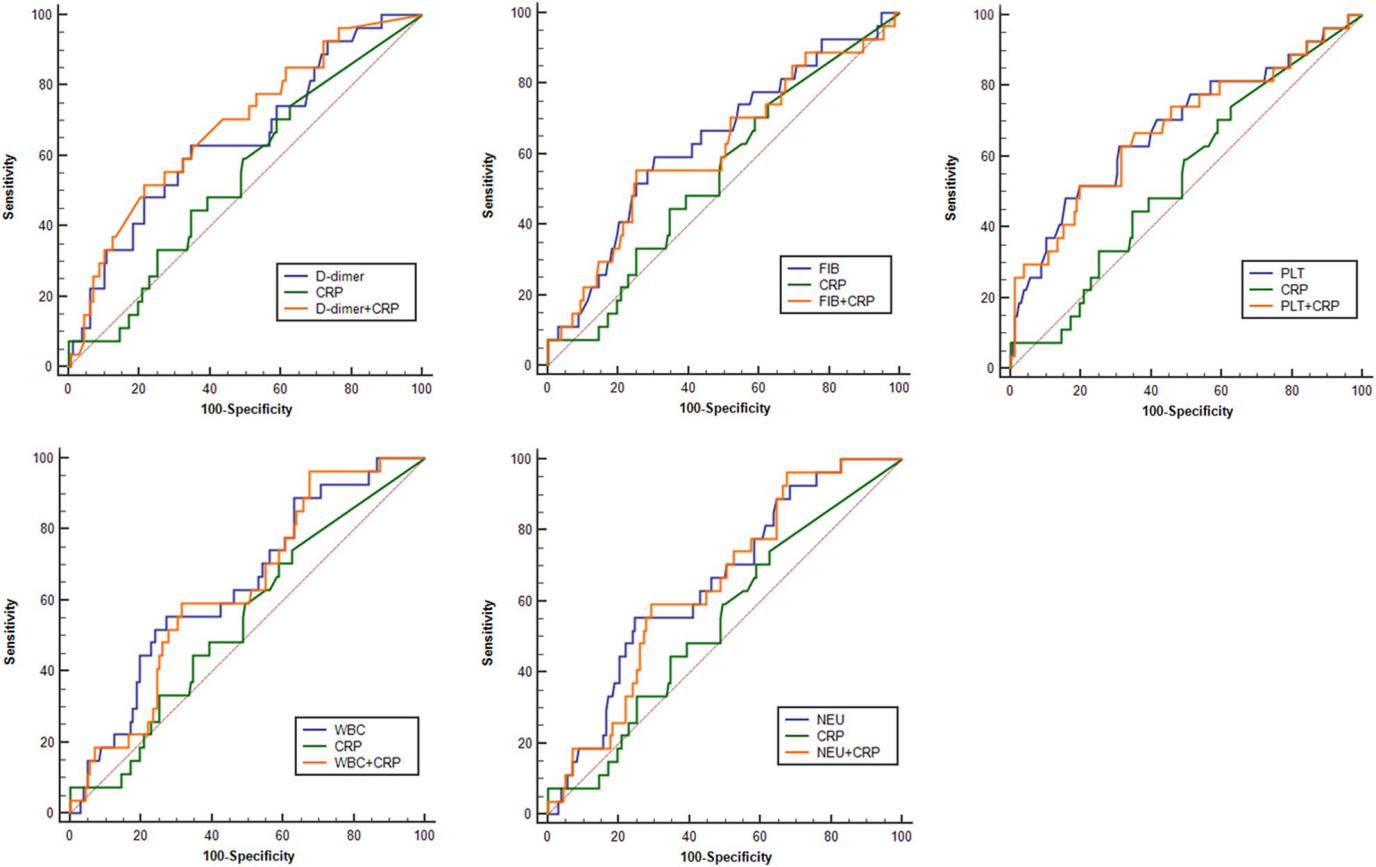
ROC curve of a single index of D-dimer, FIB, PLT, CRP, WBC, or NEU, and the combination of the single index and CRP for predicting in-hospital deaths in patients with type A AAD. ROC, receiver operating characteristic; FIB, fibrinogen; PLT, platelet; CRP, C-reactive protein; WBC, white blood cells; NEU, neutrophil; AAD, acute aortic dissection.

It can be seen from the ROC analysis that PLT had the highest specificity, while CRP was provided with the highest sensitivity. Therefore, using ROC analysis, we further evaluated the predictive value for in-hospital deaths by combining PLT or CRP with other indicators in pairs, respectively. As can be seen from Table 3, Figs. 1 and 2, the combination of FIB and PLT showed the highest predictive value for in-hospital deaths due to its greatest value of AUC among all the kinds of these combinations. The AUC of FIB + PLT combination was 0.722 [95% (0.651, 0.785)], and the sensitivity and specificity was 59.26% and 80.38%, respectively (*P* = 0.0001). The AUC of D-dimer + CRP combination was 0.686 [95% (0.614, 0.752)], and the sensitivity and specificity was 51.85% and 78.48%, respectively (*P* = 0.0008). The AUC of D-dimer + PLT combination was 0.656 [95% (0.582, 0.724)], and the sensitivity and specificity was 62.82% and 65.82%, respectively (*P* = 0.0086).

**Table 3:** Diagnostic value of combination of the single index (D-dimer, FIB, WBC, NEU) and PLT or CRP for in-hospital mortality.

Variable	AUC	SE	95% CI	Sensitivity	Specificity	*P* value
D-dimer + PLT	0.656	0.0592	0.582 to 0.724	62.96	65.82	0.0086
FIB + PLT	0.722	0.0565	0.651 to 0.785	59.26	80.38	0.0001
WBC + PLT	0.584	0.0612	0.509 to 0.656	85.19	32.91	0.1716
NEU + PLT	0.571	0.0612	0.496 to 0.644	85.19	32.28	0.2449
D-dimer + CRP	0.686	0.0556	0.614 to 0.752	51.85	78.48	0.0008
FIB + CRP	0.613	0.0625	0.539 to 0.684	55.56	74.68	0.0699
PLT + CRP	0.680	0.0622	0.608 to 0.747	51.85	80.38	0.0037
WBC + CRP	0.631	0.0533	0.557 to 0.701	96.30	32.28	0.0139
NEU + CRP	0.647	0.0517	0.573 to 0.715	59.26	70.89	0.0046

PLT, platelet; FIB, fibrinogen; WBC, white blood cells; NEU, neutrophil; CRP, C reactive protein.

**Figure 3:**
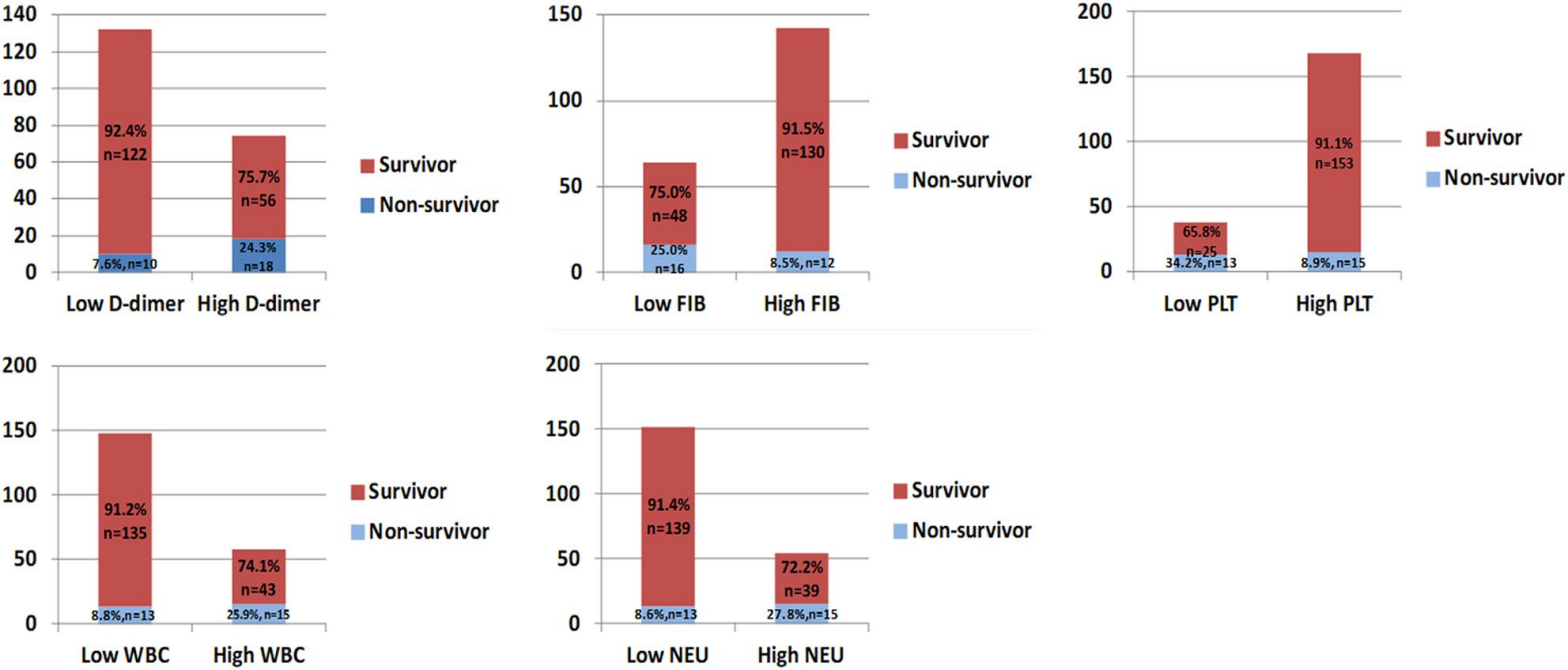
Distribution of the in-hospital mortality rate in patients with type A AAD according to categories of the indices including D-dimer, FIB, PLT, WBC and NEU. FIB, fibrinogen; PLT, platelet; WBC, white blood cells; NEU, neutrophil; AAD, acute aortic dissection.

As shown in Fig. 3, patients were divided into two groups such as high D-dimer group (≥ 10.3 mg/L) and low D-dimer group (< 10.3 mg/L) according to the optimal critical value of D-dimer., 10 of the 132 patients in the low D-dimer group (7.6%) died during hospitalization. In contrast, of the 74 patients in the high D-dimer group, 18 (24.3%) died during the hospital stays. Chi-square test showed a significant difference in mortality between the two groups (*P* = 0.002). For FIB, patients were divided into two groups such as high FIB group (> 2.12 g/L) and low FIB group (≤ 2.12 g/L) according to the optimal critical value. The percentage of patients died during hospitalization reached up to 25.0% in the low FIB group while that in the high FIB group was only 8.5%. There is a significant difference in mortality between the two groups by Chi-square test (*P* = 0.002). For PLT, patients were divided into two groups such as high PLT group (> 122 × 10^9^/L) and low PLT group (≤ 122 × 10^9^/L) according to the optimal cut-off value. 34.2% of patients died during hospitalization in the low PLT group, compared with 8.9% in the high PLT group. A significant difference in mortality was observed between the two groups by using Chi-square test analysis (*P* = 0.001). For WBC, patients were divided into two groups such as high WBC group (≥ 13.17 × 10^9^/L) and low WBC group (< 13.17 × 10^9^/L) according to the optimal critical value. The percentage of patients died during hospitalization reached up to 25.9% in the high WBC group while that in the low WBC group was only 8.8%.Chi-square test showed a significant difference in mortality between the two groups (*P* = 0.002). For NEU, patients were divided into two groups such as high NEU group (≥ 11.94 × 10^9^/L) and low NEU group (< 11.94 × 10^9^/L) according to the optimal critical value. 8.6% of patients died during hospitalization in the low NEU group, compared with 27.8% in the high NEU group. There is a significant difference in mortality between the two groups by Chi-square test (*P* = 0.001).

### Logistic regression methods

Logistic regression methods were further used to analyze the independent risk factors for postoperative in-hospital deaths of patients with type A AAD. Univariate Logistic regression analysis showed that the *P* values of D-dimer, FIB, PLT, WBC and NEU were all less than 0.1, which may be risk factors for postoperative in-hospital deaths of patients with type A AAD (Table 4). In order to exclude the influence of possible confounding factors, multivariate logistic stepwise regression analysis was conducted to identify the independent risk factors. It was found that WBC and D-dimmer were independent risk factors for postoperative in-hospital deaths of patients with type A AAD.

**Table 4:** Predictors of in-hospital mortality in patients with type A AAD by logistic regression.

Variable	Univariable			Multivariable		
	OR	95% CI	*P* value	OR	95% CI	*P* value
Age	1.106	0.752–1.625	0.610			
Sex	0.895	0.315–2.546	0.835			
BMI	1.180	0.95–1.466	0.135			
WBC	1.742	1.095–2.772	0.019	1.645	1.017–2.659	0.042
Hb	1.013	0.601–1.708	0.961			
CRP	1.069	0.837–1.364	0.593			
D-dimer	1.530	1.123–2.085	0.007	1.471	1.075–2.014	0.016
FIB	0.615	0.388–0.975	0.039			
PLT	0.988	0.978–0.997	0.013			
NEU	1.108	1.011–1.213	0.028			

BMI, body mass index; WBC, white blood cells; Hb, hemoglobin; CRP, C reactive protein; FIB, fibrinogen; PLT, platelet; NEU, neutrophil.

## Discussion

Type A AAD is one of the commonest diseases in the emergency treatment for cardiovascular surgery. Early identification of risk factors in patients with type A AAD may help to reduce the mortality risk of these patients. At present, several risk factors including older age, cardiac tamponade, hypotension, myocardial ischemia, acute renal failure, limb ischemia, neurological deficits, and mesenteric ischemia have been recognized as independent predictors of in-hospital death in AAD^1^, but these risk factors still cannot meet the needs of clinical practice. We found that WBC and D-dimer of AAD patients on admission were closely associated with increased risks of in-hospital mortality. Both preoperative D-dimer and WBC were identified as independent risk factors of in-hospital deaths for patients with type A AAD.

D-dimer is a specific end-product derived from degradation fragments of cross-linked fibrin. When the coagulation system is activated, there is a large number of thrombus forming in the body, and thus leading to the hyperactivity of fibrinolytic system as well as the increase in D-dimer production. Previous studies have confirmed that the levels of D-dimer may be useful in risk stratification of patients with suspected aortic dissection if it is used within the first 24 h following the onset of symptom^6–8^**.** After the occurrence of aortic wall injury in patients with AAD, a large quantity of tissue factors is released into blood, which may subsequently activate the coagulation system. Coagulation activation could eventually lead to the hyperreactivity of fibrinolytic system followed by the elevation of D-dimer. In recent years, many studies have also confirmed that D-dimer is associated with poor clinical prognosis of patients with dissection. Ohlmann et al. reported that D-dimer levels were correlated with the number of segment of dissected aorta and tended to be higher in AAD patients of DeBakey I type than those in both DeBakey II and DeBakey III types^9^. The mechanism by which elevated levels of D-dimer may account for the increased risk of deaths in patients with AAD is unclear. However, it was reported that in patients with acute aortic syndrome, the level of D-dimer is significantly higher in patients with AAD than that in patients with intramural hematoma, and the level of D-dimer in patients with type-A aortic dissection was also significantly higher than that in patients with type-B aortic dissection^10^. These evidences above all suggest that D-dimer may be related to the size of thrombosis and the contact area between thrombosis and blood of the patients with AAD. Elevated D-dimer may indicate larger area of aortic dissection and thrombosis in the patients. Moreover, the elevated levels of D-dimer may result from the interaction between systemic inflammation and coagulation. It is generally recognized that inflammation may activate coagulation while the activation of coagulation system may also modulate inflammatory reaction.

A large number of studies have shown that inflammation is closely related to the occurrence and development of aortic dissection. For example, CRP was reported to be increased in AAD and was correlated with markers indicating ischemia^11,12^. Likewise, WBC is higher in AAD compared to aortic aneurysms and it is associated with higher mortality in AAD patients^13,14^. Inflammation reaction plays an important role in the formation of aortic dissection, because inflammation could destroy the medial layer of the aortic wall, eventually leading to dilation, dissection, or rupture of the aortic wall and after the acute onset of AAD, mechanical injury in the lesion of aorta wall can stimulate the expression of neutrophil chemotactic factor and granulocyte colony stimulating factor, thus promoting neutrophils migration, and accumulation of a large number of neutrophils in the dissection vessel wall. The release of inflammatory factors enhanced the inflammatory reaction of the adventitia following the onset of AAD, leading to further dissection of AD and rupture of the dissection^15,16^. WBC counts are the most sensitive and direct inflammatory indicators. The higher level of WBC in AAD patients on admission was correlated with the increased mortality risk, which was consistent with the conclusion of this study. In-hospital infection is also an independent risk factor for increased in-hospital deaths of patients with AD. Infection of patients during the hospital stays will aggravate the original AD condition and cause systemic metabolic disorder. Severe infection may even lead to septic shock, respiratory failure, heart failure or multiple organ failure, which dramatically increases the risk of deaths.

Our study indicated that the combination of FIB and PLT performed better in predicting postoperative in-hospital mortality of patients with type A AAD than a single biomarker of the reason for this may be that the intimal tearing of aortic dissection gives rise to the exposure of subendothelial tissue, and thus leading to the release of tissue factors and initiation of blood coagulation cascade. Large amounts of FIB and PLT are consumed during this process, resulting in the decreased levels blood FIB and PLT in the later phase. The lower level of fibrinogen would indicate the serious injury of aorta and poorer prognosis in AAD patients^17^. Therefore, the lower levels of blood FIB and PLT are, both the more severe the aortic wall injury and the worse the prognosis will be. Considering the favorable performance of blood FIB and PLT in predicting the prognosis of the patients with type-A AAD, we recommend that FIB and PLT in the blood be detected immediately after admission of patients with AAD. Large prospective cohort studies should be conducted to further investigate the role of FIB and PLT in guiding emergency treatment of type-A AAD.

## Limitations

There are some limitations in the current study. First, blood pressure and heart rate at the onset of AAD have been confirmed as factors that may affect the prognosis of patients with AAD, but these data were not included in our analysis. The reason is that most patients with AAD who were taken to our hospital for surgical treatment were transported by ambulance after receiving symptomatic treatment such as sedation, analgesia, lowering blood pressure and controlling heart rate in local hospitals. As a result, the blood pressure and heart rate in these patients on admission were all controlled within the normal limits. In addition, family members of some patients accidentally lost the original medical record data from the local hospital, which would cause problems for us to collect data about heart rate and blood pressure at the onset of the disease. Therefore, we decided to exclude the analysis on blood pressure and heart rate of patients with AAD in this study. Second, we evaluated only the relation between blood biomarkers and in-hospital mortality, but the predicting role of blood biomarkers in the long-term prognosis of AAD patients are still unknown. Third, since the patients enrolled in this study were of Chinese Han ancestry, our results need to be confirmed by other studies including patients from different ethnic groups. Finally, the sample size of our study is relatively small. And it is a retrospective rather than a prospective study, so large-scale, multi-center clinical studies are required to further confirm the application value of blood biomarkers including D-dimer, FIB, PLT, CRP, WBC, and NEU in surgical risk assessment of patients with aortic dissection.

## Conclusions

Both preoperative D-dimer and WBC in patients with type A AAD may be used as independent risk factors for in-hospital mortality of such patients. The combination of FIB and PLT may improve the accuracy of clinical prognostic assessment. Biochemical testing has a great prospect of clinical application due to its advantages such as being quick, simple, non-invasive, and cheap. Therefore, it is advisable to search for blood biomarkers of being valuable in early diagnosis and prognosis evaluation of AAD patients, so as to better guide the future clinical research and treatment strategy.

## Data Availability

All data generated or analyzed during this study are included in this published article.

## Abbreviations

AAD: Acute aortic dissection
CRP: C-reactive protein
FIB: Fibrinogen
NEU: Neutrophil
PLT: Platelet
ROC: Receiver operating characteristic
WBC: White blood cells
